# RNase H activities counteract a toxic effect of Polymerase η in cells replicating with depleted dNTP pools

**DOI:** 10.1093/nar/gkz165

**Published:** 2019-03-08

**Authors:** Alice Meroni, Giulia Maria Nava, Eliana Bianco, Lavinia Grasso, Elena Galati, Maria Cristina Bosio, Daria Delmastro, Marco Muzi-Falconi, Federico Lazzaro

**Affiliations:** Dipartimento di Bioscienze, Università degli Studi di Milano, via Celoria 26, 20133 Milano, Italy

## Abstract

RNA:DNA hybrids are transient physiological intermediates that arise during several cellular processes such as DNA replication. In pathological situations, they may stably accumulate and pose a threat to genome integrity. Cellular RNase H activities process these structures to restore the correct DNA:DNA sequence. Yeast cells lacking RNase H are negatively affected by depletion of deoxyribonucleotide pools necessary for DNA replication. Here we show that the translesion synthesis DNA polymerase η (Pol η) plays a role in DNA replication under low deoxyribonucleotides condition triggered by hydroxyurea. In particular, the catalytic reaction performed by Pol η is detrimental for RNase H deficient cells, causing DNA damage checkpoint activation and G2/M arrest. Moreover, a Pol η mutant allele with enhanced ribonucleotide incorporation further exacerbates the sensitivity to hydroxyurea of cells lacking RNase H activities. Our data are compatible with a model in which Pol η activity facilitates the formation or stabilization of RNA:DNA hybrids at stalled replication forks. However, in a scenario where RNase H activity fails to restore DNA, these hybrids become highly toxic for cells.

## INTRODUCTION

The accuracy of genome duplication is mainly guaranteed by the high fidelity of replicative DNA polymerases that insert the correct deoxyribonucleotide respecting the base pairing with the template. Besides discriminating among the different bases, replicative polymerases have also to choose the right sugar moiety (1). In doing so, they are challenged by the intracellular physiologically high concentration of ribonucleotides (rNTPs), which exceed deoxyribonucleotides (dNTPs) by over a hundredfold (2). Specific amino acid residues shape the steric gate in the nucleotide binding site, driving DNA polymerases to select dNTPs, which lack an oxygen at the 2′ carbon of the sugar compared to rNTPs (1). Nonetheless, during DNA replication, a significant number of ribonucleotides is introduced into the nascent strand (2). In yeast, at least 1 rNTP is incorporated every 1000 dNTPs, making rNTPs the most frequent non-canonical nucleotides introduced into the genome (3). Genomic rNMPs play an important physiological role in mismatch repair (4–6) but, if not promptly removed from DNA, they also promote replication stress and genome instability (7–11).

Chromosome embedded ribonucleotides are usually processed by RNase H enzymes, which contribute to the re-establishment of the correct DNA sequence (3): RNase H1 cleaves RNA:DNA hybrids constituted by at least four consecutive ribonucleotides; RNase H2 processes both single and multiple embedded ribonucleotides (12). Noteworthy, dysfunction of the RNase H2 complex is a primary cause of the Aicardi-Goutières syndrome, a rare interferonopathy that mainly affects the brain (13).

Genomic ribonucleotides can also be subjected to mutagenic processing by Topoisomerase 1, leading to the formation of short deletions and ultimately double-strand breaks (14–16). In general, the impaired removal of ribonucleotides leads to severe consequences, as their persistence in DNA distorts the helix structure (17–19) and therefore affects DNA transactions.

Ribonucleotide incorporation is further increased when the cellular dNTP pools are reduced, such as following treatment with the ribonucleotide reductase inhibitor hydroxyurea (HU) (8). Indeed, RNase H deficient cells are hypersensitive to HU (7–9). During replication, genomic rNMPs are not efficiently bypassed by replicative DNA polymerases (7,20–22) and cells rely on post-replication repair mechanisms to overcome these blocks (7). Upon HU treatment, template switching or Pol ζ-mediated translesion synthesis (TLS) are essential to bypass rNMPs in the DNA template and to complete genome duplication (7).

Intriguingly, we found that the increased sensitivity to HU observed in yeast cells lacking RNase H activities (*rnh1Δ rnh201Δ*) is almost totally dependent on the TLS polymerase η (*RAD30*).

Pol η belongs to the Y-family polymerases and has a major role in the bypass of several adducts that halt the progression of replication forks. This versatile polymerase is known for its excellent ability in bypassing thymidine adducts and 8-oxo-guanines in an error-free manner (23–25). In humans, defects in the gene encoding Pol η (*POLH*) lead to the onset of the *xeroderma pigmentosum* variant (XP-V) genetic syndrome (26), characterized by high incidence of skin cancer and sunlight sensitivity, due to the inability to bypass bulky lesions (27). Besides the TLS function, human Pol η has been implicated in class switch recombination (28,29) and common fragile sites (CFSs) stability (30–33). Moreover, yeast Pol η was reported to be recruited at replication forks upon replication stress induction (34). Different studies reported that yeast and human Pol η efficiently utilize rNTPs and extend RNA primers *in vitro* (35–37).

In this work, we describe the involvement of Pol η in genome replication when dNTP pools are low. In particular, we report that in these conditions Pol η catalytic activity becomes harmful when ribonucleotides cannot be removed. The enhanced ribonucleotide incorporation, through a Pol η steric gate mutant (38), further exacerbates this phenotype. In the absence of RNase H activities capable of processing consecutive ribonucleotides, Pol η activity leads to the activation of the DNA damage checkpoint and to cell cycle arrest within a single round of replication in the presence of low dNTPs. We demonstrate that the toxic activity of Pol η is not dependent upon ribonucleotides already present in the template DNA and that RNase H enzymes are essential to resolve the toxic structures promoted by Pol η. We propose a model where Pol η actively participates to DNA replication in low dNTPs conditions. Pol η ability to incorporate rNMPs or extend RNA stretches could be crucial to enable full-genome duplication during replication stress at the expense of an accumulation of longer stretches of consecutive ribonucleotides in the genome. In wild type cells, thanks to RNase H, this is a tolerable compromise. However, this activity of Pol η becomes highly toxic when RNase H enzymes are defective and cells cannot restore the correct DNA composition and structure.

## MATERIALS AND METHODS

### Yeast strains, plasmids, media and growth conditions

All the strains used in this work are listed in Supplementary Table S1 and are derivatives of the W303 *RAD5*^+^ background. Strains were generated by standard genetic procedures. Deletions were obtained by one-step PCR (39). RNase H2 mutant strains (*rnh201‐P45D‐Y219A* and r*nh201‐D39A*) were obtained by crossing, starting from *MATa sgs1::HIS3 rnh201-D39A* and *MATa sgs1::HIS3 rnh201-P45D-Y219A* (40).

For the indicated experiments, cell cultures were grown at 28°C in YEP medium (1% yeast extract, 2% peptone) containing 2% glucose (YEPD), 2% raffinose (YEPR), or 2% galactose and 2% raffinose (YEPRG). For strains carrying plasmids, cells were grown in Synthetic-Complete (SC) medium supplemented with appropriate sugar(s) and nutrients to maintain the selection. The concentration of drugs/chemicals and their addition to the medium are indicated in the figures and their respective legends.

Hydroxyurea was purchased from US Biological (Salem, MA, USA), Auxin (IAA), Doxycycline (DOXY) and MMS were purchased from Sigma (Saint Louis, MO, USA).

The pEGUh6-RAD30 (*GAL1-6xHIS-RAD30*) and pEGUh6-rad30 D155A D156A (*GAL1-6xHIS-rad30-D155A-E156A*) plasmids were kindly provided by T.A. Kunkel and are described in (41). pFL166.4 (*GAL1-6xHIS -rad30-F35A*) was obtained by site-directed mutagenesis (QuikChange Site-Directed Mutagenesis Kit Thermo Fisher Scientific, Waltham, Massachusetts USA) on pEGUh6-RAD30 using oligos 3′-ACA TAG ATA TGA ATG CCT TTG CTG CAC AGG TTG AGC AGA TGC G-5′ and 3′-CGC ATC TGC TCA ACC TGT GCA GCA AAG GCA TTC ATA TCT ATG T-5′, and then verified by DNA sequencing.

The pFL160.1 plasmid carrying the *rnhB*-3xminiAID-HA under TetOFF promoter (42) was prepared as follows. The *rnhB* gene was amplified from MG1655 *E. coli* strain using primers 3′- TTA ACA TCG ATA GCG GCC GCA TGA TCG AAT TTG TTT ATC CGC ACA CG-5′ and 3′- GAC TTT TGA CAA GAA ACC ATG GAC GCA AGT CCC AGT GCG C-5′. The 3X-miniAID sequence was amplified from the plasmid BYP7432 (43) using primers 3′-GCG CAC TGG GAC TTG CGT CCA TGG TTT CTT GTC AAA AGT C-5′ and 3′-TGC AGG GCC CTA GCG GCC GCT CAC GCA TAG TCA GGA ACA TCG TAT GGG TAT TTA TAC ATT CTC AAG TCT A-5′. The two amplicons were purified and then digested with NotI and PshAI restriction enzymes. Ligation reactions were performed with NotI-digested pCM185 (42). The sequence of the insert and its junction regions was then verified by DNA sequencing (Eurofins Scientific, Luxembourg). Restriction enzymes were provided by New England Biolabs (Ipswich, MA, USA).

### Drop test assays

Logarithmically growing yeast cultures were diluted at 2 × 10^6^ cells/ml. A series of 10-fold dilutions were prepared, and 10 μl drops were spotted on YEP or selective plates, supplemented with the appropriate sugar and the indicated drugs. Pictures were taken after incubation at 28°C for 2–4 days.

### Sensitivity assay

Exponentially growing cells were synchronized in the G1 phase by adding α-factor (4 μg/ml) (Primm, Milano, Italy). Upon appropriate dilutions, 100 CFU of each strain were plated on YEPRG ± 25 mM HU. After 4 days of incubation, the number of grown colonies was counted and normalized. The standard error of the mean (SEM) was calculated on three independent experiments.

### SDS-PAGE and western blot

TCA protein extracts were prepared, and an equal amount of each sample was separated by SDS-PAGE (44). Western blottings were performed with anti-Rad53 (kind gift by C. Santocanale), anti-HIS-tag (70796-3 Novagen, Merck, Darmstadt, Germany) or anti-miniAID-tag (MBL International, Woburn, MA, USA (43)) or anti-Pgk1 (22c5d8 Abcam, Cambridge, UK) antibodies, using standard techniques.

### FACS analysis

Cells were fixed in 70% ethanol and treated with RNase A and proteinase K. DNA was stained with Sytox Green (Thermo Fisher Scientific, Waltham, MA, USA), and cell cycle distribution was estimated by cytofluorimetric analysis with a FACScan (BD Biosciences, San Josè, CA, USA). Data were plotted using the FlowJo^®^ Software.

### Ribonucleotide incorporation assay

The assay was performed as described in details in (45). Briefly, genomic DNA was isolated using Y-DER (Thermo Fisher Scientific, Waltham, Massachusetts USA) extraction kit according to manufacturer's instruction and treated with *Escherichia* *coli* RNase HII (NEB, Ipswich, Massachusetts USA), which introduces nicks at every ribonucleotide-containing site. The nicks were then used to radioactively label the site with DNA Polymerase I (NEB, Ipswich, MA, USA) in the presence of unlabeled dA/-dT/-dGTP and α-^32^P-dCTP (Perkin Elmer, Waltham, MA, USA). Labeled genomic DNA was separated by agarose gel electrophoresis in the presence of ethidium bromide, imaged under UV light and quantified with ImageLab software (Bio-Rad). The gel was successively dried, and the radioactive signal was detected by autoradiography using a Typhoon FLA 7000 (GE Healthcare Life Sciences, Marlborough, MA, USA) and quantified with ImageQuant software (GE Healthcare Life Sciences, Marlborough, MA, USA). The radioactive signal of each sample was normalized on total genomic DNA measured by ethidium bromide staining. The ratio between *in-vitro* RNHII-treated and the untreated sample was expressed as fold change respect to the control sample. The mean of four different experiments ± SEM is reported in the figure.

## RESULTS

### DNA polymerase η is responsible for HU-induced cell lethality in the absence of RNase H activities

**Figure 1.**
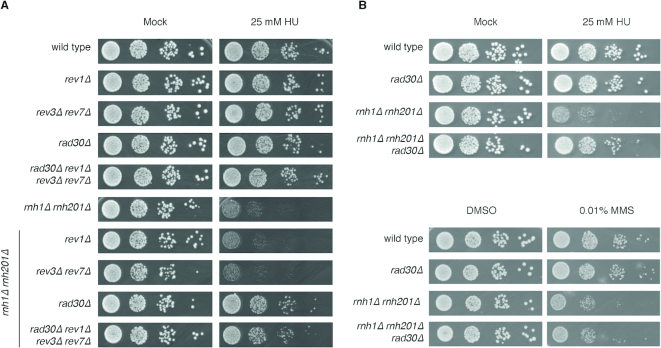
Removal of Pol η rescues the HU sensitivity of cell lacking RNase H activities. 10-fold serial dilutions of the indicated strains were plated on (**A**) YEPD and YEPD + 25 mM HU and (**B**) on YEPD, YEPD + 25 mM HU and YEPD + 0.01% MMS. Plates were incubated at 28°C and pictures were taken after 3 days. Results are representative of four biological replicates.

Yeast cells lacking both RNase H activities (*rnh1Δ rnh201Δ*) are sensitive to several genotoxic and replication stress-inducing agents, such as MMS, CPT, and HU (7,46,47). In particular, following HU treatment, the Post Replication Repair (PRR) pathway becomes essential for cell survival, and TLS polymerase ζ is critical to help replicative polymerases bypass the ribonucleotides persisting in the template strand of genomic DNA (7). Unexpectedly, we found that the simultaneous loss of all yeast TLS polymerases, Pol ζ, Pol η and Rev1 (*rev3Δ rev7Δ rad30Δ rev1Δ*) almost completely suppresses the HU sensitivity phenotype of *rnh1Δ rnh201Δ* cells (Figure 1A). By analyzing the individual contribution of each TLS polymerase, we found that the suppression of HU sensitivity is almost completely dependent upon the loss of DNA polymerase η (encoded by *RAD30* gene) (Figure 1A). Furthermore, Pol η affects *rnh1Δ rnh201Δ* cell viability specifically following HU treatment, but not upon treatment with other genotoxic agents impacting on S phase, like MMS (Figure 1B).

### Low doses of HU induce a Pol η-dependent DNA damage checkpoint activation and mitotic arrest in RNase H deficient cells

We have previously shown that upon treatment with low doses of HU, RNase H lacking cells exhibit DNA damage checkpoint activation and cell cycle arrest at G2/M phase (7). Given the involvement of Pol η in the HU sensitivity of *rnh1Δ rnh201Δ* cells, we tested its contribution to such checkpoint activation.

**Figure 2.**
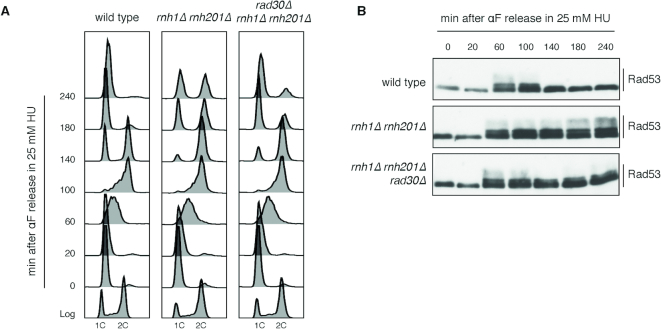
Pol η is responsible for the DNA damage checkpoint activation and G2/M arrest of RNase H deficient cells in low doses of HU. Exponentially growing cells were synchronized in G1 phase by α-factor addition (4 μg/ml) and released in 25 mM HU; α-factor (10 μg/ml) was re-added to the medium 90 min after the release to block cells in the next G1 phase. (**A**) Cell cycle progression was followed by flow cytometry (FACS) measuring DNA content (1C, 2C) at the indicated time points. (**B**) Rad53 phosphorylation was analyzed by western blotting of total cell extracts with anti-Rad53 antibodies. Results are representative of four biological replicates.

The phosphorylation state of the checkpoint effector kinase Rad53 was used as a readout for DNA damage checkpoint (DDC) activation, while cell cycle profiles were analyzed by flow cytometry (48,49). Cells were synchronized in G1 phase with α-factor, released in 25 mM HU, and collected at the indicated time points. At low doses of HU, wild type cells exhibit a mild and transient activation of the DDC in S phase; the checkpoint response is then switched off, and cell cycle progression continues (Figure 2A and B). In *rnh1Δ rnh201Δ* cells, however, Rad53 phosphorylation persists after S phase and cells cannot complete the cell cycle and entry into the next G1 phase is delayed (Figure 2A and B). Consistently with their reduced viability, a fraction of these cells remains in G2/M with 2C DNA content (Figure 2A); no cell cycle defects or Rad53 phosphorylation are observed in the absence of HU (Supplementary Figure S1). Intriguingly, deletion of *RAD30* rescues almost completely all the HU-induced phenotypes observed in RNase H mutants. Indeed, most *rnh1Δ rnh201Δ rad30Δ* cells dephosphorylate Rad53 and exhibit an almost wild type kinetics of cell cycle progression (Figure 2A and B).

It is worth noting that even in the absence of *RAD30* a small proportion of cells remain blocked in G2/M with Rad53 phosphorylated, suggesting that other polymerases may contribute to the HU-induced toxicity.

### Pol η acts at HU-stressed replication forks

Hydroxyurea is known to induce replication forks stalling and the formation of ssDNA gaps, which are filled in late S phase mainly by TLS polymerases (50–52). Yeast Pol η was reported to be recruited to the proximity of replication origins during HU-induced stress (34,53). In human cells, Pol η actively participates to CFSs replication, possibly by substituting Pol δ (31–33), and forms nuclear foci upon hydroxyurea exposure (54). Thus, we asked whether the toxic activity of Pol η occurs at the fork during DNA replication under low dNTPs conditions or post-replication, during gap refilling in late S phase/G2.

In order to address this question, we exploited a yeast strain lacking endogenous *RAD30*, where Pol η could be conditionally overexpressed at different times following a transient exposure to HU. We first tested whether an acute exposure to high levels of HU had the same effect as the chronic low dose HU treatment used so far. We exposed α-factor synchronized cells for two hours to 200 mM HU, which was then washed out, rapidly restoring physiological dNTPs level (55–57). Upon release in the cell cycle in the absence of HU, *rnh1Δ rnh201Δ* cells exhibited similar, albeit milder, phenotypes as those reported following a chronic exposure to 25 mM HU (compare Supplementary Figure S2 and Figure 2). Importantly, even though the drug was removed from the medium when cells resumed cell cycle progression, the activation of the DDC and the G2/M cell cycle arrest in *rnh1Δ rnh201Δ* cells was still dependent on Pol η (Supplementary Figure S2).

**Figure 3.**
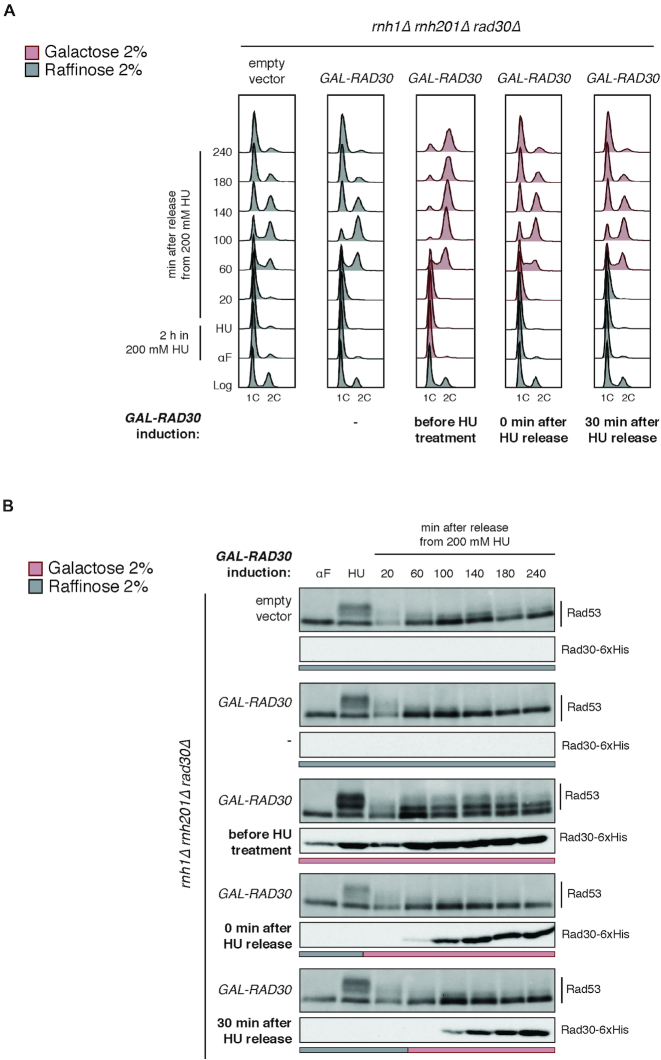
Pol η exerts its toxic activity at HU-stressed DNA replication forks. Cells were grown in SC-URA Raffinose 2%, arrested with α-factor (4 μg/ml) and released from the G1 block by transferring them in SC-URA Raffinose 2% with 200 mM HU for 2 h. HU was then washed out and cells were transferred to fresh medium to allow completion of the cell cycle. α-factor (10 μg/ml) was re-added 90 min after HU wash out to block cells in the next G1 phase. Media were supplemented with Galactose 2% to induce *RAD30* expression, when indicated (red). (**A**) Cell cycle progression was followed by flow cytometry (FACS) measuring DNA content (1C, 2C) at the indicated time points. (**B**) Rad53 phosphorylation and Rad30-6xHis expression were analyzed by western blotting of total cell extracts using appropriate antibodies. Results are representative of three biological replicates.

To determine when Pol η exerts its toxic activity, we synchronized *rnh1Δ rnh201Δ rad30Δ* cultures, and we induced *RAD30* overexpression, before, immediately after or 30′ after the acute HU treatment. In the first case, cells were subjected to nucleotide depletion in the presence of Pol η. In the other samples, cells experienced the HU-induced stress in the absence of Pol η, which was expressed only immediately or 30′ after HU removal. Protein levels were verified by western blotting; cell cycle progression and DDC activation were monitored as previously described. Intriguingly, we observed a strong accumulation of G2/M arrested cells and Rad53 phosphorylation only when Pol η was concomitantly present with hydroxyurea during DNA replication (Figure 3, before HU treatment). In contrast, when Pol η was induced after the removal of HU, most cells did not exhibit cell cycle progression defects or DDC activation (Figure 3, 0 min after HU release, 30 min after HU release). These data indicate that Pol η exerts its toxic effect at replication forks when they are slowed down and challenged by low dNTPs conditions.

### Pol η toxicity is independent of ribonucleotides embedded in the DNA replication template

Since Pol η activity becomes toxic only in the absence of RNase H, it is essential to assess how the two are related to each other. Lack of RNase H causes the genome-wide accumulation of unprocessed ribonucleotides, which could stimulate a Pol η activity that becomes toxic when dNTP pools have been depleted. On the other hand, Pol η may be recruited at the HU-stressed replication forks generating a product that becomes toxic in the absence of RNase H. In the first scenario, Pol η would be toxic in the absence of RNase H due to the presence of rNMPs in the template strand. In the second case, the inability to process ribonucleotides would be lethal only after Pol η action. To distinguish between these two hypotheses, we evaluated the effects of unprocessed rNMPs within a single cell cycle. We produced a heterologous conditional system, where RNase H2 can be inactivated at different times in a synchronous culture. A yeast strain was generated where we expressed *E. coli rnhB* gene fused to the AID-degron, so that the chimeric protein is degraded following the addition of auxin (IAA) (42,43). The fusion was cloned under the TetOFF promoter so that its expression can be switched off by addition of doxycycline (DOXY). The RNase H2 activity provided by the RnhB-AID fusion protein complemented the simultaneous loss of RNase H1 and H2 in yeast cells, suppressing almost completely the HU sensitivity and the accumulation of ribonucleotides incorporated in genomic DNA (Supplementary Figure S3). Moreover, expression of the *rnhB-AID* construct did not alter cell cycle progression of an otherwise wild type strain (Supplementary Figure S4).

**Figure 4.**
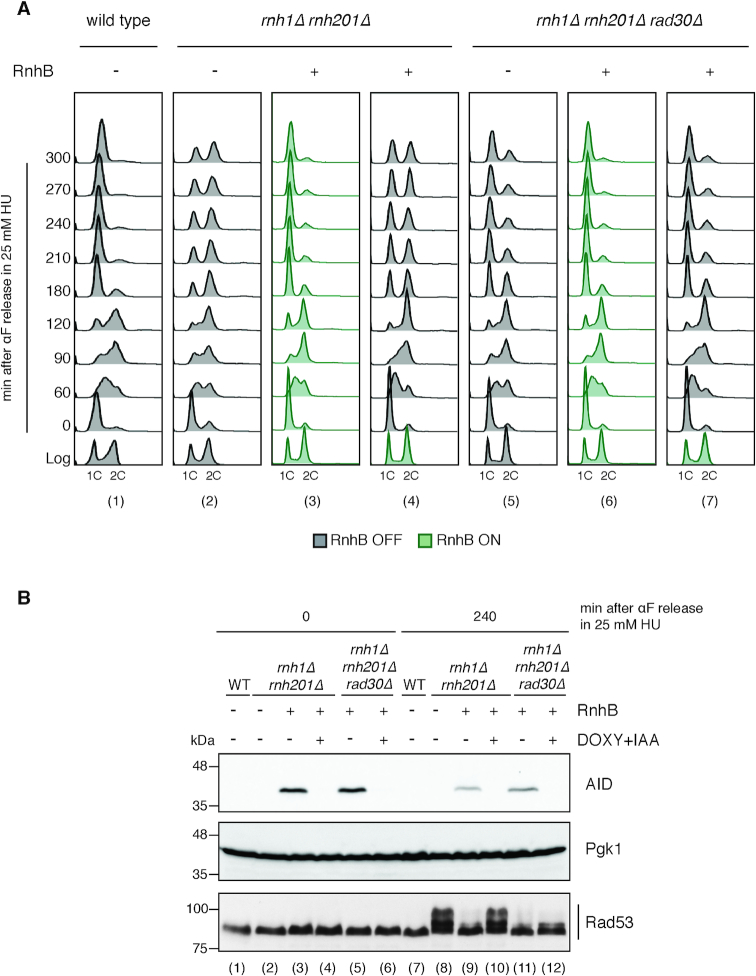
Pol η generates toxic intermediates at stressed replication forks in HU. Exponentially growing cells were synchronized in SC-TRP glucose 2% by α-factor addition (4 μg/ml) and released in YEPD + 25 mM HU. α-factor (10 μg/ml) was re-added 90 min after the release. RnhB was expressed (RnhB ON, in green) and was depleted as needed by addition of 10 μg/ml Doxycycline (DOXY) and 0.5 mM Auxin (IAA) (RnhB OFF, in gray). All the strains host the OsTIR1 gene integrated at the *URA3* locus. (**A**) Cell cycle progression was followed by flow cytometry (FACS) measuring DNA content (1C, 2C) at the indicated time points. (**B**) Rad53 phosphorylation, RnhB-AID and the Pgk1 loading control were analyzed by western blotting of total cell extracts using appropriate antibodies. Results are representative of three biological replicates.

*rnh1Δ rnh201Δ* cells carrying the conditional *rnhB-AID* construct were grown in the presence of the RnhB (ON), allowing efficient removal of pre-existing ribonucleotides, and arrested with α-factor. RnhB was then turned off (OFF) in α-factor, before the release in 25 mM HU impeding the removal of newly incorporated ribonucleotides. Cells were collected at different time points; cell cycle progression was monitored by FACS analysis (Figure 4A), and DDC activation was evaluated by western blotting (Figure 4B). In the presence of RnhB activity, despite being exposed to a low dose of HU, strains mutated in *RNH1* and *RNH201* progressed throughout the cell cycle and normally reached the next G1 phase, similarly to wild type cells (Figure 4A, panels 1, 3, 6). In these conditions, Rad53 hyperphosphorylation was almost completely absent (Figure 4B, lanes 7, 9, 11). These results further confirm that RnhB-AID efficiently complements the absence of yeast endogenous RNase H activities. When RnhB was turned off before the release in HU, *rnh1Δ rnh201Δ* cells experienced the first round of DNA replication in the presence of hydroxyurea and in the absence of RNase H activities (Figure 4A, lanes 4 and 7). In these conditions, contrary to what observed when RnhB was expressed, cells arrested at G2/M within the first cell cycle (Figure 4A, panel 4) with hyperphosphorylated Rad53 (Figure 4B, lane 10), with a phenotype similar to that of *rnh1Δ rnh201Δ* cells carrying the empty plasmid (Figure 4A, panel 2 and Figure 4B, lane 8). Moreover, deletion of *RAD30* suppressed both the cell cycle arrest and the DDC activation (Figure 4A, panel 7 and Figure 4B, lane 12).

These findings suggest that when Pol η acts at replication forks in low dNTPs, it poses a lethal threat to cells if RNase H activity is missing. This argues against the involvement in the toxic effect of Pol η of pre-existing rNMPs in the template strand.

### Pol η toxicity is exerted via its catalytic activity, and it is enhanced by a steric gate mutation that increases rNTPs incorporation

**Figure 5.**
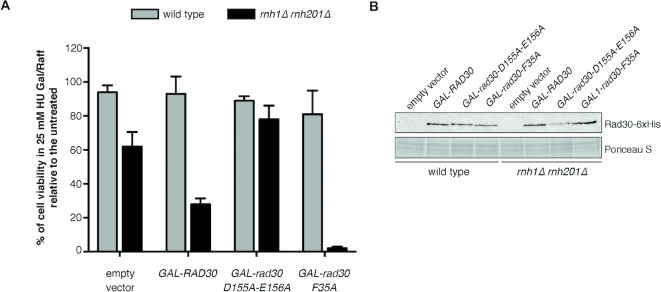
Pol η toxicity depends on its catalytic activity and it is exacerbated if its ribonucleotide-incorporation activity is increased. (**A**) Cells were grown in SC-URA Raffinose 2% and arrested with α-factor. Cultures were then appropriately diluted and plated on SC–URA supplemented with Galactose 2% and Raffinose 2%, either with or without 25 mM HU. Colonies were counted from at least three plates after 4 days at 28°C. Histogram bars represent the ratio between colonies counted on plates with and without hydroxyurea. Error bars represent the standard error of the mean (SEM), calculated on three independent experiments. (**B**) Overexpression levels of wild type and mutant versions of Rad30 were analyzed by western blot with anti-HIS antibodies 4 h after Galactose induction; equal loading was verified by Ponceau S staining.

To characterize the mechanism underlying Pol η toxicity, we investigated the involvement of its catalytic activity. HIS-tagged wild type (*RAD30*), catalytic-dead (*rad30-D155A-E156A*) alleles or a steric gate mutant with enhanced ribonucleotide incorporation activity (*rad30-F35A*) (38,58) were overexpressed in yeast cells under the control of the *GAL1/10* promoter. Overexpression was necessary because the steric gate mutation although increasing rNTPs incorporation, strongly reduces the catalytic activity of the enzyme. Indeed, a *rad30-F35A* mutant expressed at the endogenous level is catalytically deficient and UV sensitive, while the UV sensitivity is suppressed when overexpressed, likely because enough Pol η catalytic is present (Supplementary Figure S5A and B). Cell viability in 25 mM HU was measured and it is reported in Figure 5A. Overexpression of either wild type or mutant forms of Pol η was achieved at similar levels (Figure 5B) and did not noticeably affect the viability of RNase H wild type cells both in untreated or HU-treated conditions (Figure 5A, grey bars, and Supplementary Figure S5C). In cells lacking RNase H, on the other hand, overexpression of wild type Pol η exacerbated the sensitivity to HU, linking the extent of the toxic activity to the level of Pol η in cells (Figure 5A, black bars). Conversely, overexpression of the catalytic-dead mutant (*rad30-D155A-E156A*) suppressed the HU-induced cell lethality restoring viability to levels comparable to those observed in RNase H proficient cells, implying that Pol η catalytic activity is responsible for the HU sensitivity. This was confirmed by the steric gate mutant *rad30-F35A*, which confers enhanced ribonucleotide incorporation activity (37,38) and caused a drastic increase in HU sensitivity to *rnh1Δ rnh201Δ* cells, suggesting that the incorporation of ribonucleotides mediated by Pol η is the cause of cell lethality (Figure 5A, black bars).

**Figure 6.**
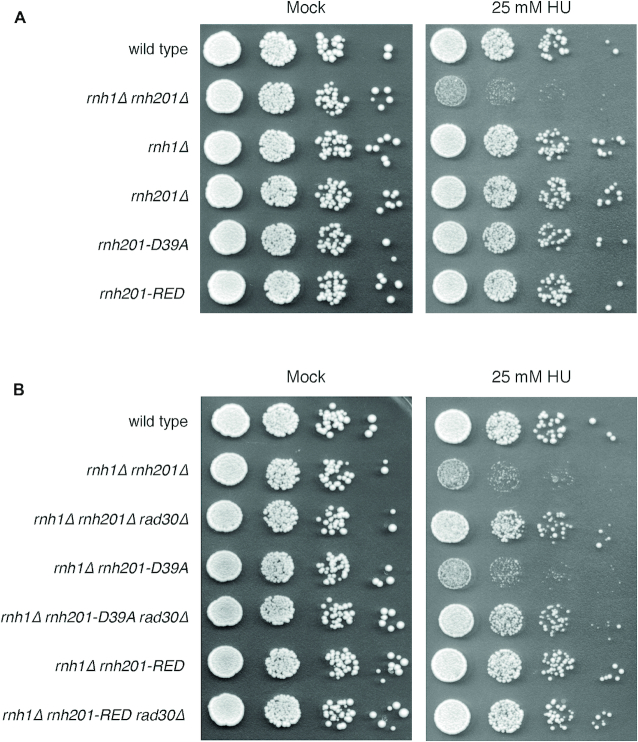
HU sensitivity is due to the failure to process multiple consecutive ribonucleotides. 10-fold serial dilutions of the indicated strains were plated on YEPD and YEPD + 25 mM HU and incubated at 28°C. Pictures were taken after 2 days of incubation. Results are representative of two biological replicates. The D39A mutation inactivates the catalytic function of RNase H2 while the separation of function mutant *rnh201-RED* allows the removal of stretches of rNMPs but is impaired in the removal of single rNMPs.

To examine whether unprocessed genomic ribonucleotides were responsible for the HU-induced lethality, we analyzed the HU sensitivity imparted by individual *RNH1* (RNase H1) and *RNH201* (RNase H2) deletions, by an RNase H2 catalytic-dead mutant (*rnh201-D39A*) and by a separation of functions mutant *rnh201-RED* (*rnh201-P45D-Y219A*), which has been reported to be able to process multiple consecutive ribonucleotides, while being impaired in the removal of single rNMPs (40) (Figure 6A). The same mutations were then combined with the deletion of *RAD30* to gain an insight into the type of toxic product generated by Pol η (Figure 6B).

The results reveal that HU becomes lethal only when cells cannot process consecutive ribonucleotides (*rnh1Δ rnh201Δ* or *rnh1Δ rnh201-D39A*) but not if consecutive ribonucleotides can be processed by RNase H1 (*rnh201Δ*) or if the ability of RNase H2 to act on multiple rNMPs is preserved (*rnh1Δ rnh201-RED*). Intriguingly, the data shown in Figure 6B also shows that Pol η is toxic only if multiple ribonucleotides cannot be processed, suggesting that when the dNTP pools are decreased Pol η activity may promote the incorporation of stretches of ribonucleotides in the genome.

## DISCUSSION

Ribonucleotides are massively incorporated during DNA replication by DNA polymerases, and, if not repaired, they cause genome instability. Depletion of the dNTP pools by hydroxyurea inhibits replicative DNA synthesis. Interestingly, exposure of *rnh1Δ rnh201Δ* cells to even low levels of HU leads to DNA damage checkpoint activation and G2/M arrest. TLS DNA polymerase η is well known for its roles in lesion bypass for the effective completion of DNA synthesis, following exposure to DNA damaging agents (23,24).

**Figure 7.**
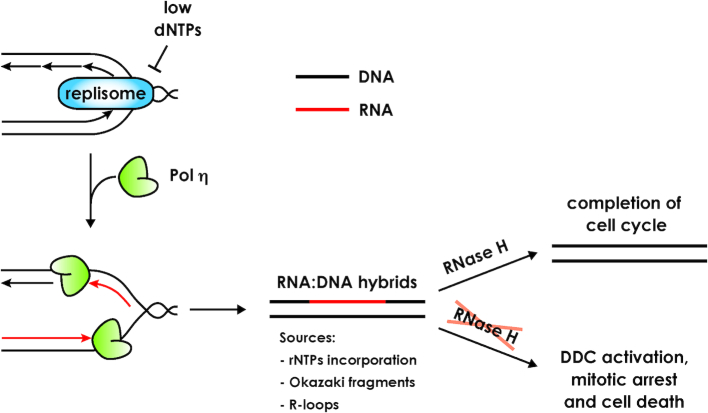
Proposed model of Pol η function during hydroxyurea-mediated fork stalling. Replication forks are stalled by dNTP pools depletion caused by HU, allowing Pol η recruitment. Pol η activity promotes RNA:DNA hybrids formation by either direct incorporation of ribonucleotides in the newly synthesized strand or incorrect Okazaki fragments maturation or R-loops stabilization. These hybrids are later processed by RNase H activities, allowing completion of the cell cycle. In the absence of RNase H, RNA:DNA hybrids are not removed from the genome and lead to DNA damage checkpoint activation, cell cycle arrest and ultimately cell death.

We describe a novel involvement of Pol η during genome duplication under replication stress conditions. In particular, we propose that, when canonical DNA polymerases are challenged by deoxyribonucleotide depletion, Pol η facilitates replication fork progression and chromosomal replication favoring the inclusion of consecutive ribonucleotides in newly synthesized genomic DNA. Pol η is suitable for this function thanks to its ability to extend DNA or RNA primers with ribonucleotides (37,38), unlike replicative DNA polymerases, which exhibit lower incorporation capability and are blocked by dNTPs shortage (2,3). Even though this function may be relevant to complete DNA replication and tolerate temporary decreases in the dNTP pools, it presents a potentially lethal challenge when RNase H activities are absent, and chromosome embedded ribonucleotides are excessively accumulated (Figure 7). The genomic sites bound by Pol η in HU-treated cells have been previously mapped and some of them correspond to ARSs (34) that are actively replicated in cells exposed to 200 mM HU (34,53). Moreover, human Pol η is required for S phase progression in hydroxyurea, and its activity induces apoptotic cell death (36,54).

In this work we show that:

- When cellular dNTP pools are depleted by exposure to low doses of HU, Pol η catalytic activity promotes the formation of some structures that become toxic only if RNase H activities are lost, while no effect is detected in cells expressing RNase H functions. This strongly suggests a role for ribonucleotides in the Pol η-dependent HU-induced lethality.
- Such toxic products are generated when Pol η is active at replication forks stressed by dNTPs depletion, while no problem arises if Pol η acts later in S phase, once dNTP pools have been replenished.
- The formation of the toxic products by Pol η does not depend upon residual rNMPs in the template strand, but it involves ribonucleotides incorporated during the S phase with low dNTP pools, and it elicits a DNA damage checkpoint and G2/M arrest within the same cell cycle.
- The sensitivity to HU, and thus the formation of the toxic products, is greatly enhanced if cells express a Pol η variant with enhanced rNTPs incorporation ability. Moreover, the HU-induced lethality depends upon the inability in the processing of multiple consecutive ribonucleotides.

Physical detection of multiple rNMPs embedded in genomic DNA has proven to be very hard to tackle with the available technology; no groups to our knowledge were able to address this problem yet. Even if we cannot directly measure the presence of Pol η-dependent multiple ribonucleotides, the data summarized above very strongly support the hypothesis that the phenotypes reported in this work are related to RNA stretches embedded in genomic DNA, which are accumulated only when Pol η is active and the dNTP pools are depleted. Such RNA stretches may arise from the direct incorporation of rNTPs by Pol η, from a Pol η-dependent stabilization of R-loops or impairment of Okazaki fragments processing, consistently with recent evidence showing that Pol η could take part in the replication of the lagging strand (59). Remarkably, following replication with low dNTP pools and active Pol η, if ribonucleotides cannot be processed cells die because they cannot divide (Figure 7). Careful kinetic analyses reveal that, in these conditions, the DDC is switched off after S phase and re-activated when cells reach 2C DNA content in G2/M. Taken together, these observations may correlate RNA:DNA hybrids with chromosome segregation. We propose that RNA:DNA hybrids may compromise chromosomes structure (17–19,60) generating problems that are detected in G2/M and trigger the DDC. Such hybrids may also lead to mutagenic repair mechanisms, such as the one dependent on Top1, leading to deletions and chromosome breaks (15,16). We can also envision that the alteration of chromosome structure due to newly incorporated ribonucleotides may affect particular regions, e.g. centromeres, essential for proper cell division. This hypothesis is supported by the observation that deletion of the Spindle Assembly Checkpoint factor *MAD2* partially rescues the HU sensitivity, cell cycle arrest of cells lacking RNase H and persistent DNA damage checkpoint activation (Supplementary Figure S6).

As mentioned above, such scenario may be conserved in higher eukaryotes as well. This mechanism resembles the one reported in human cells for Chromosome Fragile Sites (CFSs) replication. CFSs are peculiar sequences that assume non-B DNA structures that block replication forks (61,62). Here, Pol η substitutes Pol δ and carries on DNA synthesis, avoiding the formation of breaks and preventing under-replicated DNA from entering mitosis (30–33). As in hydroxyurea, Pol η may act when replication forks are stalled.

In conclusion, this study unravels a novel role of TLS polymerase η and provides evidence of its contribution to the preservation of genome integrity. In the future, it will be interesting to investigate how Pol η can participate to repair synthesis in environments where dNTP pools are physiologically low, such as in neuronal cells.

## Supplementary Material

Supplementary Data

## Notes

Present address: Alice Meroni, Edward A. Doisy Department of Biochemistry and Molecular Biology, Saint Louis University School of Medicine, 1100 South Grand Blvd St. Louis, MO 63104.

Present address: Eliana Bianco, Institute of Biochemistry, ETH Zurich, Otto-Stern-Weg 3, CH-8093 Zurich, Switzerland.
