# Supplementary material for: RNase H activities counteract a toxic effect of Polymerase η in cells replicating with depleted dNTP pools
Source: Nucleic Acids Res. 2019 Mar 8;47(9):4612–23. doi: 10.1093/nar/gkz165 (PMC6511917; doi:10.1093/nar/gkz165)
Supplement: Supplementary Data [file gkz165_supplemental_file.pdf]

Figure S1

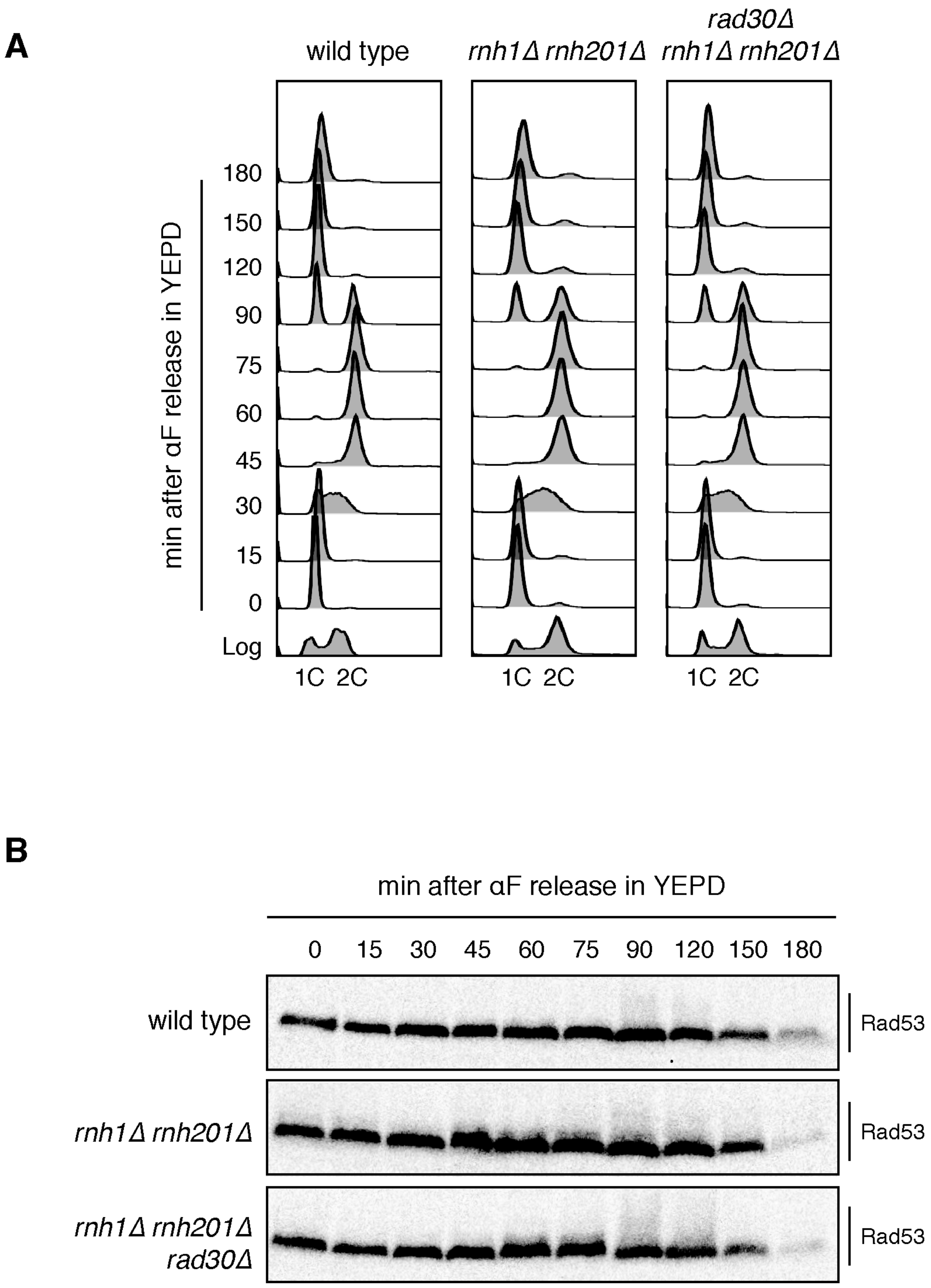

Figure S1. Strains lacking RNase H activity do not exhibit cell cycle delay and DDC activation in untreated conditions.

(A-B) Exponentially growing cells were synchronized in G1 phase by α-factor addition (4 µg/mL) and released in fresh YEPD medium. α-factor (10 µg/mL) was re-added to the medium 90 min after the release to block cells in the next G1 phase. **(A)** Cell cycle progression was followed by flow cytometry (FACS) measuring DNA content (1C, 2C) at the indicated time points. **(B)** Rad53 phosphorylation was analyzed by western blotting of total cell extracts using anti-Rad53 antibodies. Results are representative of two biological replicates.

Figure S2

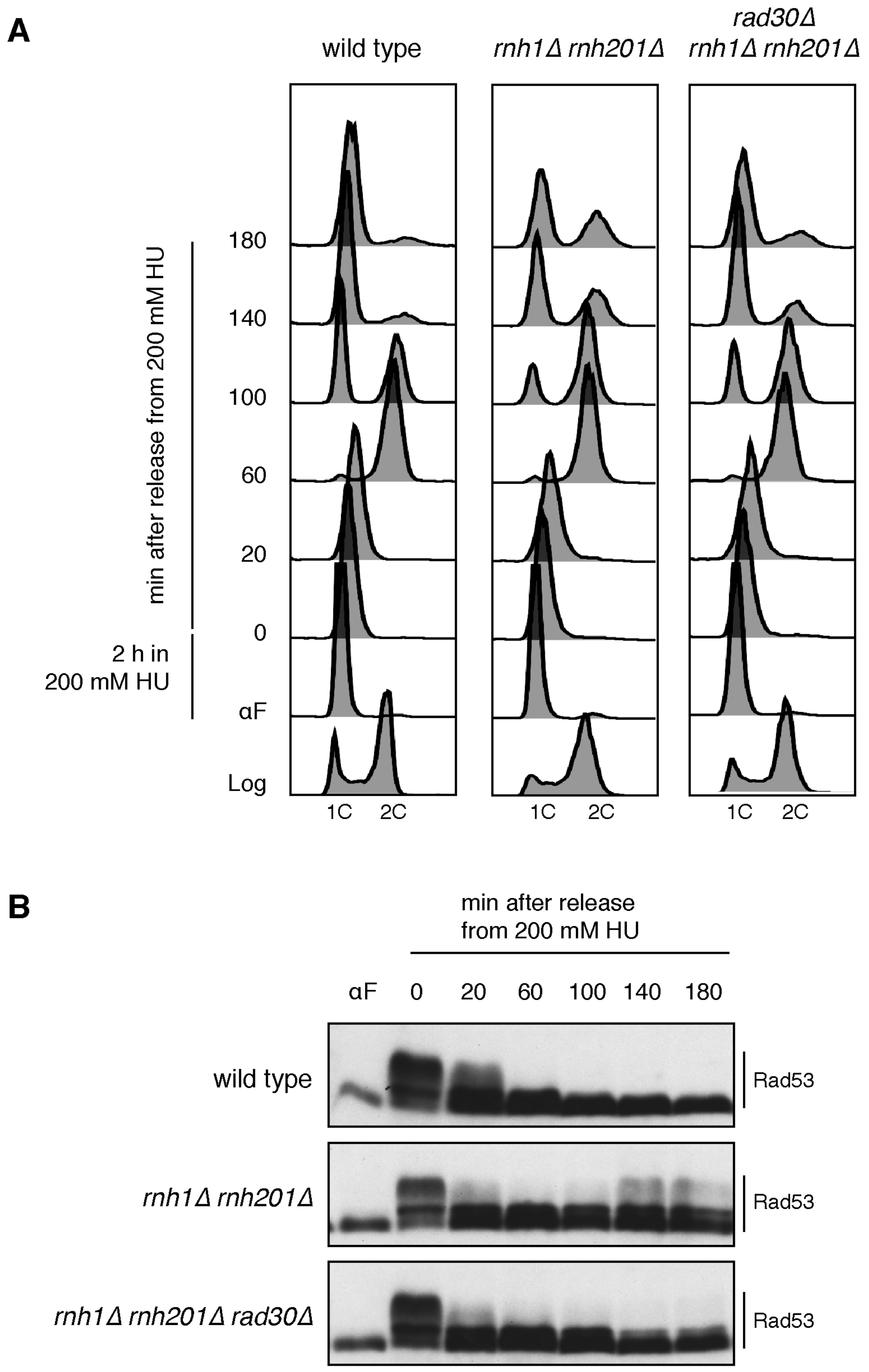

Figure S2. In RNase H deficient cells Pol η promotes DDC activation and G2/M arrest, even after acute HU exposure.

(A-B) Exponentially growing cells were synchronized in G1 phase by α-factor addition (4 µg/mL) and released from the G1 arrest in 200 mM HU for 2 hours. HU was then washed out and cells were transferred to fresh medium to allow completion of the cell cycle. α-factor (10 µg/mL) was then re-added 40 min after the HU wash out to block cells in the next G1 phase. **(A)** Cell cycle progression was followed by flow cytometry (FACS) measuring DNA content (1C, 2C) at the indicated time points.
(B) Rad53 phosphorylation was analyzed by western blotting of total cell extracts using anti-Rad53 antibodies. Results are representative of three biological replicates.

Figure S3

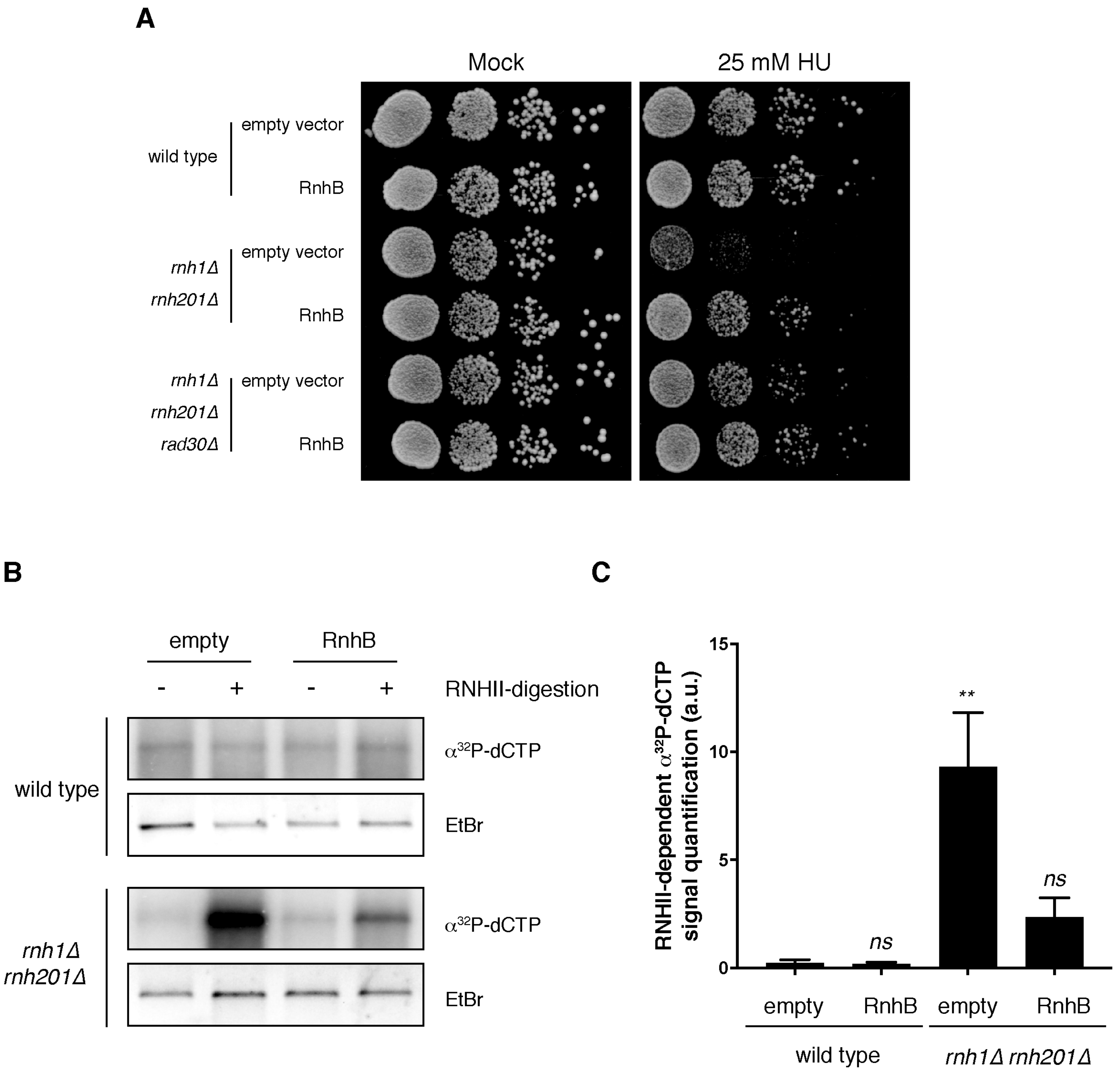

Figure S3. In yeast cells lacking endogenous RNase H enzymes, bacterial RnhB restores the ribonucleotides removal activity.

(A) 10-fold serial dilutions of the indicated strains were plated on SC-TRP and SC-TRP + 25 mM HU and incubated at 28°C. RnhB-AID is constitutively expressed. Pictures were taken after 4 days of incubation. Results are representative of three biological replicates.

(B) Ribonucleotides incorporation assay on genomic DNA extracted from G1 synchronized wild type and *rnh1Δrnh201Δ* cells transformed either with the empty plasmid or the one carrying the RnhB-AID coding gene. All the strains have the *OsTIR1* gene integrated at *URA3* locus.

(C) Ribonucleotides incorporation was quantified and is expressed in arbitrary units as fold change of the RNHII treated sample respect to the untreated. All samples were normalized on the corresponding ethidium bromide signal. The error bars represent the SEM of four independent experiments. Student t-test is performed relative to the wild type [empty] sample (*ns* = non significative, ** = P ≤ 0.01).

Figure S4

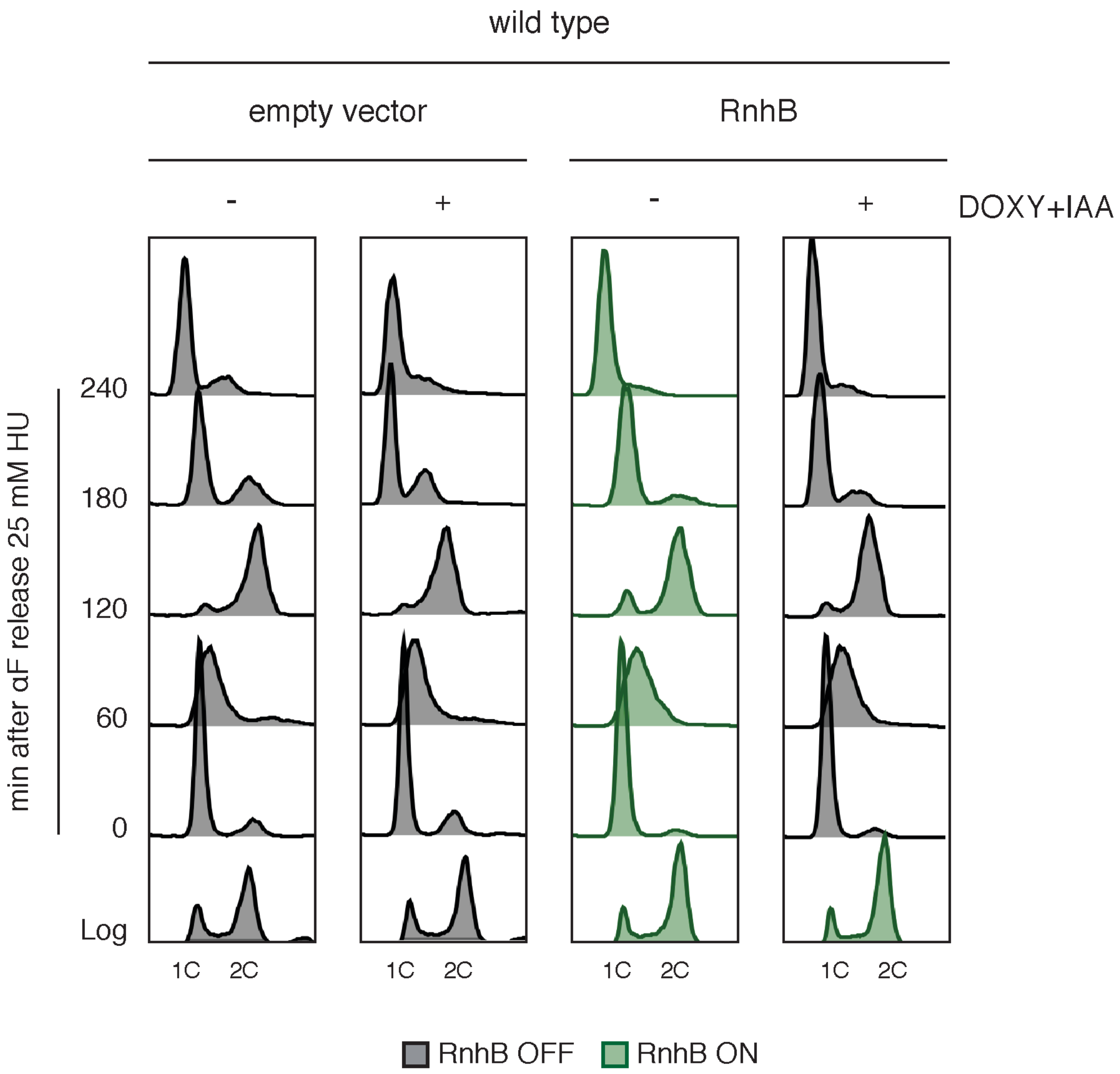

Figure S4. Expression of RnhB does not affect cell cycle progression in wild type cells. Exponentially growing cells were synchronized in SC-TRP Glucose 2% by α-factor addition (4 µg/mL) and released in YEPD + 25 mM HU. α-factor (10 µg/mL) was re-added 90 min after the release. RnhB-AID was expressed (RnhB ON, in green) and was depleted as needed by addition of 10 µg/mL Doxycycline (DOXY) and 0.5 mM Auxin (IAA) (RnhB OFF, in grey). All the strains carry the *OsTIR1* gene integrated at *URA3* locus. Cell cycle progression was followed by flow cytometry (FACS) measuring DNA content (1C, 2C) at the indicated time points. Results are representative of three biological replicates.

Figure S5

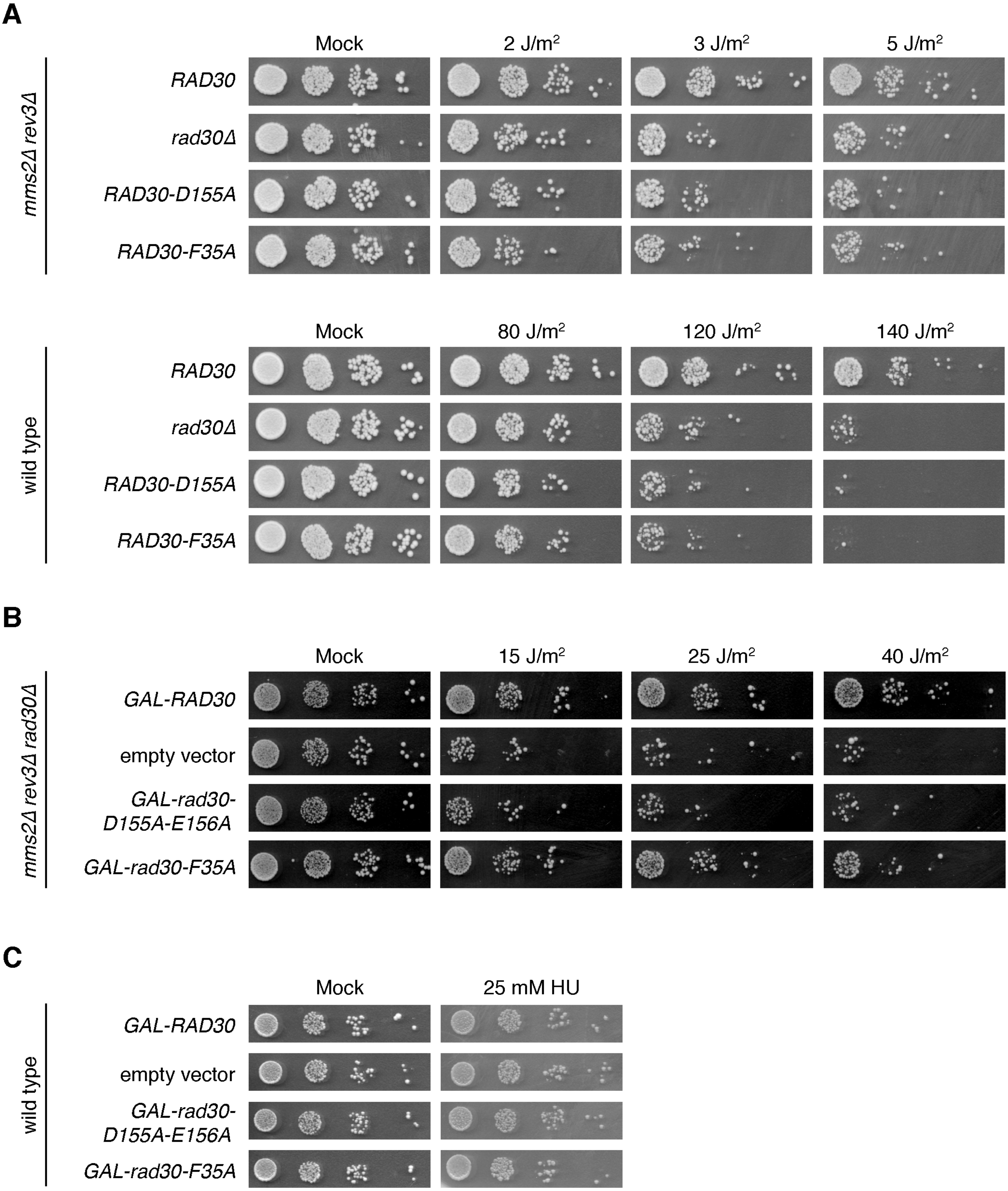

Figure S5. *rad30-F35A* mutant complements UV sensitivity only if overexpressed and wt cell viability is not affected by overexpression of Rad30, *rad30-D155A-E156A* and *rad30-F35A*.
(A)10-fold serial dilutions of the indicated strains were plated on YEPD and UV irradiated.Pictures were taken after 2 days of incubation at 28°C. Results are representative of two biological replicates.
(B)10-fold serial dilutions of the indicated strains were plated on SC-URA, supplemented with Raffinose 2% and Galactose 2% and UV iradiated. Pictures were taken after 3 days of incubation at 28°C. Results are representative of two biological replicates.
(C)10-fold serial dilutions of the indicated strains were plated on SC-URA and SC-URA + 25 mM HU, supplemented with Raffinose 2% and Galactose 2%. Pictures were taken after 3 days of incubation at 28°C. Results are representative of three biological replicates.

Figure S6

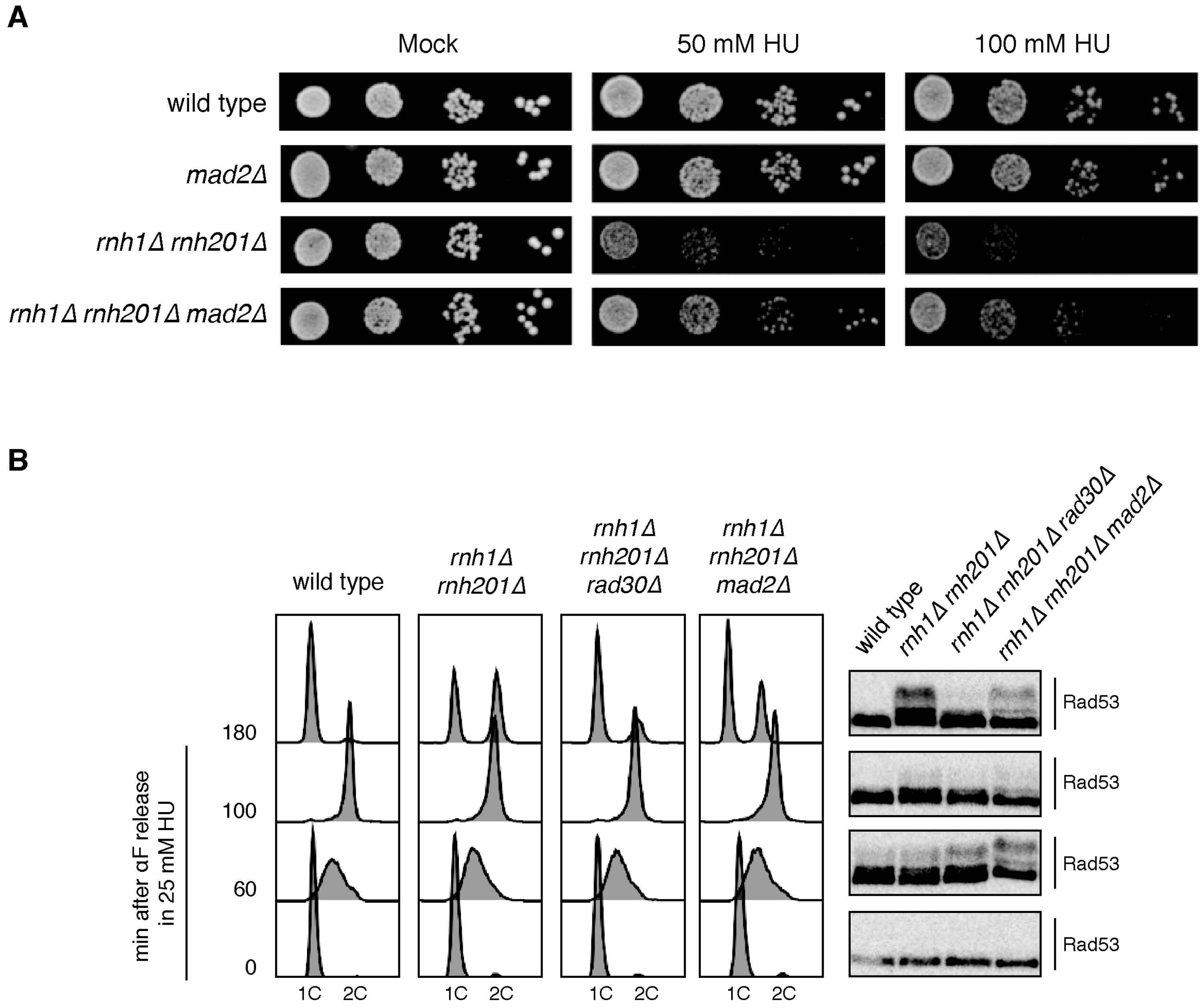

Figure S6. The toxic effect of Pol η is partly dependent upon the spindle assembly checkpoint factor Mad2.
(A) 10-fold serial dilutions of the indicated strains were plated on YEPD and YEPD + 50 mM or 100 mM HU Pictures were taken after 2 days of incubation. **(B)** Exponentially growing cells were synchronized in G1 phase by α-factor addition (4μg/ml) and released in 25 mM HU. α-factor was re-added to the medium 90 min after the release to block cells in the next G1 phase. In the left panel cell cycle progression was followed by flow cytometry (FACS) measuring DNA content (1C, 2C) at the indicated time points. In the right panel Rad53 phosphorylation was analyzed by western blotting of total cell extracts using anti-Rad53 antibodies. Results are representative of two biological replicates.

Table S1

Strain	Genotype	Ref.
SY2080	*W303 MATa ade2-1 trp1-1 leu2-3,112 his3-11,15 ura3-1 can1-100 RAD5*	M. Foiani
YFL1213	*(SY2080) MATa rnh1::HIS3 rnh201::KANMX6*	(7)
YFL1773	*(SY2080) MATa rnh1::HIS3 rnh201::KANMX6 rad30::TRP1*	This Study
YSS21	*(SY2080) MATa rad30::KANMX6*	(63)
YMG1082	*(SY2080) MATa rad30::KANMX6 rev1::KANMX6 rev3::TRP1 rev7::HIS3*	(63)
YFL1271	*(SY2080) MATa rad30::KANMX6 rev1::KANMX6 rev3::TRP1 rev7::HIS3 rnh1::HIS3 rnh201::KANMX6*	(7)
YSS17	*(SY2080) MATa rev1::KANMX6*	This Study
YFL2485	*(SY2080) MATa rev1::HPH rnh1::HIS3 rnh201::KANMX6*	This Study
YMG1096	*(SY2080) MATa rev3::TRP1 rev7::HIS3*	This Study
YFL1389	*(SY2080) MATa rev3::TRP1 rev7::HIS3 rnh1::HIS3 rnh201::KANMX6*	This Study
YFL1419	*(SY2080) MATa + pRS426*	This Study
YFL1420	*(SY2080) MATa + pEGUh6-RAD30 [GAL1-6XHIS-RAD30-URA3]*	This Study
YFL1421	*(SY2080) MATa + pEGUh6-rad30 D155A D156A [GAL1-6XHIS-rad30-D155A-E156A-URA3]*	This Study
YFL2567	*(SY2080) MATa + pFL166.4 [GAL1-6XHIS-rad30-F355A-URA3]*	This Study
YFL1422	*(SY2080) MATa rnh1::HIS3 rnh201::KANMX6 + pRS426*	This Study
YFL1423	*(SY2080) MATa rnh1::HIS3 rnh201::KANMX6 + pEGUh6-RAD30 [GAL1-6XHIS-rad30-URA3]*	This Study
YFL1424	*(SY2080) MATa rnh1::HIS3 rnh201::KANMX6 + pEGUh6-rad30 D155A D156A [GAL1-6XHIS-rad30-D155A-E156A-URA3]*	This Study
YFL2569	*(SY2080) MATa rnh1::HIS3 rnh201::KANMX6 + pFL166.4 [GAL1-6XHIS-rad30-F355A-URA3]*	This Study
YFL2591	*(SY2080) MATa ura3::ADH1-OsTIR1-9MYC:URA3 + pCM185 [TRP]*	This Study
YFL2596	*(SY2080) MATa ura3::ADH1-OsTIR1-9MYC:URA3 rnh1::HIS3 rnh201::KANMX6 + pCM185 [TRP]*	This Study
YFL2603	*(SY2080) MATa ura3::ADH1-OsTIR1-9MYC:URA3 rnh1::HIS3 rnh201::KANMX6 rad30::LEU2 + pCM185 [TRP]*	This Study

YFL2592	*(SY2080) MATa ura3::ADH1-OsTIR1-9MYC:URA3 + pFL160.1 [rnhB-AID-HA-TRP]*	This Study
YFL2598	*(SY2080) MATa ura3::ADH1-OsTIR1-9MYC:URA3 rnh1::HIS3 rnh201::KANMX6 + pFL160.1 [rnhB-AID-HA-TRP]*	This Study
YFL2604	*(SY2080) MATa ura3::ADH1-OsTIR1-9MYC:URA3 rnh1::HIS3 rnh201::KANMX6 rad30::TRP1 + pFL160.1 [rnhB-AID-HA-TRP]*	This Study
YFL3045	*(W303 RAD5) MATa sgs1::HIS3 rnh201-D39A*	(38)
YFL3044	*(W303 RAD5) MATa sgs1::HIS3 rnh201-P45D-Y219A*	(38)
YFL3049/2D	*(SY2080) MATa rnh201-D39A*	This Study
YFL3047/3A	*(SY2080) MATa rnh201-P45D-Y219A*	This Study
YFL1208/2D	*(SY2080) MATa rnh1::HIS3*	(7)
YFL1191/4B	*(SY2080) MATa rnh201::KANMX6*	(7)
YFL3068/2A	*(SY2080) MATa rnh201-D39A rnh1::HIS3*	This Study
YFL3071/3D	*(SY2080) MATa rnh201-D39A rnh1::HIS3 rad30::TRP1*	This Study
YFL3062/2A	*(SY2080) MATa rnh201-P45D-Y219A rnh1::HIS3*	This Study
YFL3066/4A	*(SY2080) MATa rnh201-P45D-Y219 rnh1::HIS3 rad30::TRP1*	This Study
YFL1229/1A	*(SY2080) MATa rnh1::HIS3 rnh201::KANMX6 mad2::TRP1*	This Study
YFL1228/2A	*(SY2080) MATa mad2::TRP1*	This Study
YFL3180	*(SY2080) MATa mms2::HPH rev3::TRP1 rad30::KANMX6 + pRS426*	This Study
YFL3182	*(SY2080) MATa mms2::HPH rev3::TRP1 rad30::KANMX6 + pEGUh6-RAD30 [GAL1-6XHIS-rad30-URA3]*	This Study
YFL3184	*(SY2080) MATa mms2::HPH rev3::TRP1 rad30::KANMX6 + pEGUh6-rad30 D155A D156A [GAL1-6XHIS-rad30-D155A-E156A-URA3]*	This Study
YFL3186	*(SY2080) MATa mms2::HPH rev3::TRP1 rad30::KANMX6 + pFL166.4 [GAL1-6XHIS-rad30-F355A-URA3]*	This Study
YFL3188	*(SY2080) MATa mms2::HPH rev3::TRP1 RAD30-13MYC:KANMX6*	This Study
YFL1496/4a	*(SY2080) MATa mms2::HPH rev3::TRP1 rad30::KANMX6*	This Study
YFL3177/17d	*(SY2080) MATa mms2::HPH rev3::TRP1 rad30-D155A-13MYC:KANMX6*	This Study

YFL3174/12a	*(SY2080) MATa mms2::HPH rev3::TRP1 rad30-F35A-13MYC:KANMX6*	This Study
YSS16	*(SY2080) MATa RAD30-13MYC:KANMX6*	This Study
LS237	*(SY2080) MATa rad30-D155A-13MYC:KANMX6*	(34)
YFL2945	*(SY2080) MATa rad30-F35A-13MYC:KANMX6*	This Study
